# Cannabidiol for Mucosal Diseases: Therapeutic Potential and Advanced Delivery Strategies

**DOI:** 10.3390/pharmaceutics18060638

**Published:** 2026-05-22

**Authors:** Bo Han, Yue Zhang, Yangmin Wang, Yue Shen, Jinping Niu, Shipo Li, Yuxi Li, Jingyu Wang, Xingyuan Ma, Wenyun Zheng

**Affiliations:** 1 Shanghai Key Laboratory of New Drug Design, School of Pharmacy, East China University of Science and Technology, Shanghai 200237, China; bohan@mail.ecust.edu.cn (B.H.); y53230064@mail.ecust.edu.cn (J.W.); 2 Shaanxi Key Laboratory of Natural Products & Chemical Biology, College of Chemistry & Pharmacy, Northwest A&F University, Yangling 712100, China; zhangyuey@nwafu.edu.cn; 3 State Key Laboratory of Bioreactor Engineering, East China University of Science and Technology, Shanghai 200237, China; y10230042@mail.ecust.edu.cn (Y.W.); y10220054@mail.ecust.edu.cn (Y.S.); y10210027@mail.ecust.edu.cn (J.N.); y10220048@mail.ecust.edu.cn (S.L.); y30230570@mail.ecust.edu.cn (Y.L.)

**Keywords:** cannabidiol, endocannabinoid system, mucosal diseases, routes of administration, delivery system

## Abstract

Cannabidiol (CBD), a major non-psychoactive phytocannabinoid, has attracted considerable attention owing to its broad therapeutic potential. Its anti-inflammatory, antimicrobial, and antitumor properties make it a promising candidate for the treatment of mucosa-associated diseases. However, the clinical translation of CBD is significantly hindered by its unfavorable physicochemical properties, particularly high lipophilicity and poor aqueous solubility, which result in low bioavailability. To overcome these limitations, the rational selection of administration routes in combination with advanced drug delivery systems tailored to disease pathophysiology is essential. Such strategies are critical for improving the stability of CBD, enhancing mucosal permeation, and enabling controlled and targeted release at diseased sites. Nevertheless, a systematic review focusing on these aspects is still lacking. This review first summarizes the relationship between CBD and the mucosal endocannabinoid system, together with its pharmacological effects. It then discusses the therapeutic potential of CBD in mucosal disorders of the digestive and respiratory systems. In addition, current administration routes and advanced delivery systems for CBD are reviewed to provide insights for future research and clinical translation. Finally, the remaining challenges associated with the clinical application of CBD and future development directions are discussed.

## 1. Introduction

*Cannabis* was domesticated in East Asia during the early Neolithic period, resulting in a long cultivation history. The earliest records of its use in China can be traced back thousands of years. Cannabinoids represent a class of bioactive compounds defined by their interaction with cannabinoid receptors in the human body. This class is primarily divided into phytocannabinoids, endocannabinoids, and synthetic cannabinoids. Phytocannabinoids constitute the terpenophenolic secondary metabolites originally discovered in cannabis. To date, more than 130 distinct cannabinoids have been identified in the plant, among which Δ^9^-tetrahydrocannabinol (THC) and cannabidiol (CBD) have been the subject of the most intensive research [1]. THC was among the first cannabinoids to be identified and remains the most renowned for its psychoactive effects, which include euphoria and hallucinations. It is a primary driver of cannabis addiction. Legally, in most countries, *Cannabis* is classified as hemp if it contains ≤0.3% THC (dry weight), and as marijuana if the THC content exceeds this threshold [2]. CBD exhibits a distinct pharmacological profile to THC. In contrast to THC, CBD does not produce intoxication, dependence, or drug-seeking behavior and is generally considered to have a more favorable tolerability profile. However, clinically relevant safety concerns, including hepatotoxicity signals and drug–drug interaction risks, have also been reported [3]. Therefore, the therapeutic promise of CBD should be considered together with its dose-, formulation-, and context-dependent safety profile.

Accumulating evidence highlights the diverse pharmacological profile of CBD, which includes anti-anxiety, anticonvulsant, antidepressant, antioxidant, anti-inflammatory, anticancer, antiemetic, and analgesic properties [4]. Advances in understanding the endocannabinoid system and other molecular targets of CBD have spurred significant progress in its application for mucosal diseases. This review therefore outlines the role of the endocannabinoid system in mucosal pathophysiology and summarizes the therapeutic effects of CBD on disorders of the digestive and respiratory systems. Furthermore, it discusses the major challenges associated with developing CBD as a drug product and proposes potential strategies to address them. A key focus is the rational selection of administration routes and delivery systems, informed by the physicochemical and pharmacological characteristics of CBD, to enable its successful clinical translation.

## 2. CBD and the Endocannabinoid System in the Mucosa

The mucosal system comprises the mucosal lining that covers the body’s internal surfaces exposed to the external environment, along with its associated accessory structures. This lining is organized into three primary layers: the epithelium, the lamina propria, and the muscularis mucosae. Key accessory components include glands, goblet cells, and specialized immune structures [5,6]. The mucosal system encompasses the mucous membranes of the gastrointestinal, respiratory, and urogenital tracts. It is critically involved in a range of physiological functions, including providing a protective barrier, facilitating secretion and lubrication, enabling the exchange and absorption of substances, and mounting immune defenses [7]. As the body’s principal gateway to the external world, the mucosal system is vulnerable to dysfunction, which can precipitate various diseases such as infections, inflammatory conditions, allergies, and cancers [8]. The health of this system is thus paramount for sustaining normal physiological function and ensuring robust defense against pathogens.

The endocannabinoid system (ECS) is a fundamental neurophysiological signaling network with versatile regulatory capabilities. It plays a critical role in a wide array of physiological processes, including the maintenance of homeostasis, nervous system function, pain modulation, emotional regulation, cognition, lipid and glucose metabolism, immune and inflammatory responses, and cell proliferation [9]. The ECS comprises three core components: endocannabinoids, their synthesizing and metabolizing enzymes, and cannabinoid receptors [10]. The two principal endocannabinoids in humans are N-arachidonoylethanolamine (AEA) and 2-arachidonoylglycerol (2-AG). Other notable endocannabinoid-like compounds include N-palmitoylethanolamine (PEA), N-oleoylethanolamine (OEA), and 2-oleoylglycerol (2-OG) [11]. Endocannabinoids are membrane-derived lipid signaling molecules that are synthesized upon demand in response to physiological stimuli, contrasting with classical neurotransmitters that are pre-formed and stored in vesicles. The biosynthesis of AEA originates from membrane phospholipids, primarily through the action of N-acylphosphatidylethanolamine phospholipase D (NAPE-PLD). Its primary route of inactivation is hydrolysis by fatty acid amide hydrolase (FAAH), yielding arachidonic acid and ethanolamine [12]. Similarly, 2-AG is principally synthesized by diacylglycerol lipase isoforms (DAGLα and DAGLβ) and degraded by monoacylglycerol lipase (MAGL) into arachidonic acid and glycerol [13,14]. Two canonical G protein-coupled receptors (GPCRs) for cannabinoids have been identified: cannabinoid receptor 1 (CB1) and cannabinoid receptor 2 (CB2) [15]. Within the mucosa, CB1 is predominantly expressed on enteric neurons, whereas CB2 is primarily located on immune cells [16,17]. Additionally, several other receptors capable of binding endocannabinoids are present in mucosal tissues, including the orphan GPCRs (GPR55, GPR119), transient receptor potential channels (TRPV1, TRPV4, TRPA1), and peroxisome proliferator-activated receptors (PPARα, PPARγ) [18,19].

**Figure 1:**
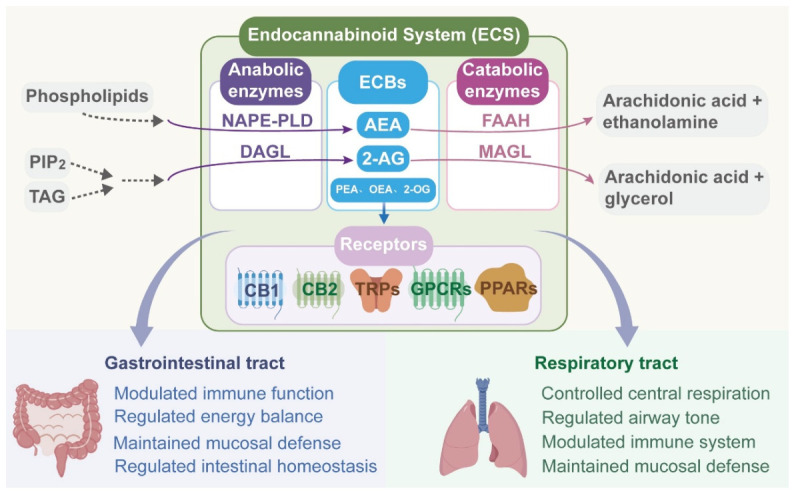
The composition of the ECS and its role in the gastrointestinal tract and respiratory tract.

**Figure 2:**
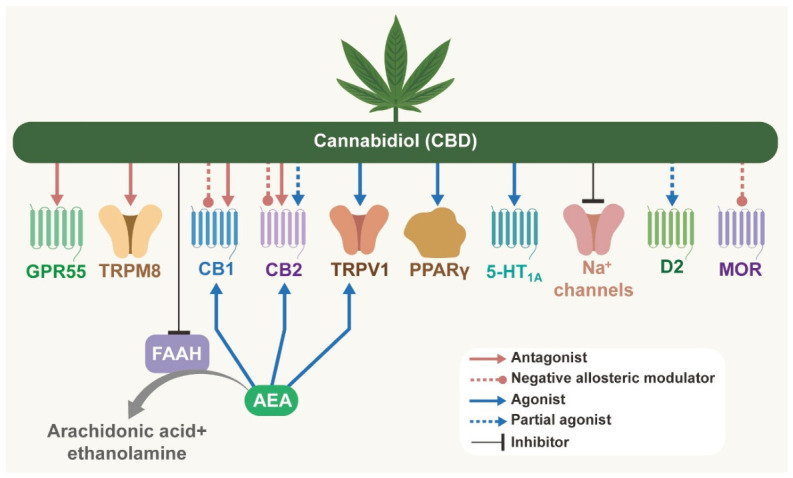
The main molecular targets of CBD and its mechanism of action mainly involve the ECS.

ECS has emerged as a promising therapeutic target for mucosal diseases [20]. For instance, AEA and 2-AG regulate appetite, energy balance, and inflammation, thereby influencing conditions like obesity, non-alcoholic fatty liver disease (NAFLD), and irritable bowel syndrome (IBS) [21]. In the respiratory mucosa, AEA has been reported to reduce transepithelial electrical resistance and increase epithelial permeability, an effect that appears to involve arachidonic acid metabolites generated during AEA metabolism [22]. Therefore, modulation of AEA metabolic pathways may influence airway epithelial barrier function; however, whether FAAH inhibition is beneficial in asthma remains to be established. Moreover, cannabinoids can suppress vagal nerve activation and cough reflexes, exerting analgesic and anti-inflammatory effects. They also modulate gastrointestinal motility, inflammation, and energy metabolism via central and peripheral activation of CB1 and CB2 [23]. Collectively, these findings underscore the diverse and pivotal roles of the ECS in maintaining mucosal homeostasis (Figure 1). The biological effects of CBD are primarily mediated through the ECS. CBD exhibits a complex polypharmacology at cannabinoid receptors: it binds with relatively low affinity to the orthosteric sites of CB1 and CB2, yet it acts as a non-competitive negative allosteric modulator at their allosteric sites [24,25,26]. Furthermore, CBD can function as a non-competitive antagonist of both receptors and may also exhibit partial agonism at CB2 [27]. Given that CBD shares molecular targets with endogenous cannabinoids, understanding its potential impact on the mucosal ECS is critically important [28]. CBD modulates endocannabinoid signaling by interacting with fatty acid-binding proteins (FABPs), which mediate the intracellular transport of AEA to its catabolic enzyme, FAAH. By binding to FABPs, CBD reduces the metabolism of AEA, thereby enhancing its tonic signaling [29]. Beyond the endocannabinoid system, CBD also engages a broad spectrum of non-cannabinoid receptors and channels, including G protein-coupled receptors (GPR18, GPR55, GPR119), µ opioid receptor (MOR), dopamine receptor 2 (D2), glycine, 5-hidroxytriptamine 1A receptor (5-HT_1A_), adenosine receptors, γ -aminobutyric acid (GABA_A_) receptor, peroxisome proliferator-activated receptors (PPARs), transient receptor potential cation channel subfamily M member 8 (TRPM8), transient receptor potential vanilloid (TRPV) channels, and voltage-gated sodium (Nav) channels [30]. The engagement of these targets activates downstream signaling pathways underlying its diverse pharmacological effects (Figure 2). Nevertheless, the precise mechanism of action of CBD in mucosal system diseases requires further elucidation.

## 3. Therapeutic Potential of CBD for Mucosal Diseases

### 3.1. Therapeutic Potential of CBD for Digestive System Diseases

Digestive system diseases, encompassing a spectrum of disorders from oral mucositis and inflammatory bowel disease (IBD) to gastrointestinal cancers, represent a major global health challenge [31]. Driven by factors such as dietary shifts, heightened life stress, environmental pollution, and population aging, their incidence continues to rise worldwide. These conditions account for over one-third of prevalent cases and contribute significantly to the overall disease burden, making them a leading driver of healthcare resource utilization and costs [32]. Despite advances in medical technology, treatment remains challenging, underscoring the urgent need for safer and more effective therapeutic agents.

#### 3.1.1. Oral Inflammatory Diseases

**Figure 3:**
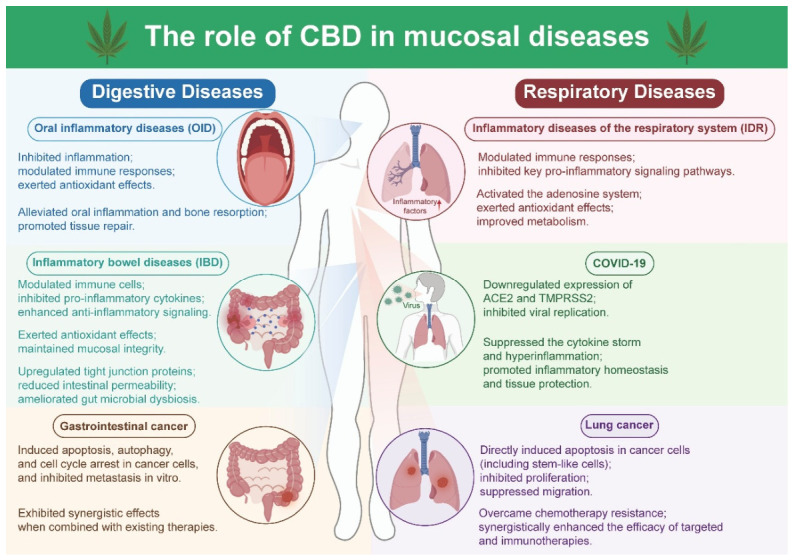
The role of CBD in mucosal diseases.

Oral inflammatory diseases, characterized by recurrent lesions and severe pain, lack effective treatment options, driving the search for novel therapeutics. CBD holds considerable promise due to its documented analgesic, anti-edema, antioxidant, and cytokine-modulating properties [33]. In a rat periodontitis model, Napimoga et al. demonstrated that CBD downregulated the RANKL/RANK axis, reduced neutrophil migration, and decreased the production of IL-1β and TNF-α, suggesting a potential role in controlling bone resorption [34]. Similarly, in a 5-fluorouracil (5-FU)-induced mouse model of oral mucositis, CBD mitigated inflammatory processes, lesion severity, and oxidative stress, while promoting tissue repair [35]. Furthermore, in human gingival mesenchymal stem cells (hGMSCs), CBD inhibited the NALP3-inflammasome pathway by reducing levels of NALP3, CASP1, and IL-18, and modulated immune activation markers—downregulating CD40, CD46, CD68, CD59, CD99, CD109, and CD151, while upregulating CD47, CD55, and CD276 [36]. Pretreating hGMSCs with CBD thus improved their molecular phenotype and reduced the risk of host immune and inflammatory responses upon transplantation. However, in a rat oral wound-healing model, CBD exerted early anti-inflammatory effects but did not significantly improve traumatic ulcer healing [37] (Figure 3). Although current clinical evidence remains limited, existing studies indicate promising efficacy of CBD in oral inflammatory conditions, warranting further investigation to clarify its mechanisms and evaluate potential adverse effects.

#### 3.1.2. Inflammatory Bowel Diseases

Inflammatory bowel disease (IBD), including ulcerative colitis (UC) and Crohn’s disease (CD), is a chronic relapsing inflammatory disorder of the gastrointestinal tract caused by interactions between genetic susceptibility, environmental factors, and dysregulated immune responses [38]. UC is characterized by continuous superficial mucosal inflammation limited to the colon, whereas CD presents with discontinuous, transmural, and segmental inflammation that may affect any part of the gastrointestinal tract [39,40]. Increasing evidence indicates that the ECS, through its anti-inflammatory, cytoprotective, and homeostatic roles, is closely involved in IBD pathogenesis and may represent a promising therapeutic target [41]. Among cannabinoids, CBD has attracted particular interest because of its anti-inflammatory and immunomodulatory properties, absence of psychoactivity, and favorable safety profile [42,43]. In preclinical models, CBD has shown protective effects against intestinal inflammation through multiple mechanisms. In DNBS-induced colitis, intraperitoneal CBD reduced colonic injury by increasing IL-10 while decreasing inducible nitric oxide synthase (iNOS) and IL-1β levels [44]. In vitro studies further suggest that CBD may protect intestinal epithelial cells by suppressing oxidative stress [45]. CBD also appears to preserve mucosal integrity, as shown in human colon explants and Caco-2 cells, where it attenuated IL-17A-mediated damage [46]. However, its anti-inflammatory efficacy varies across models, with some studies reporting significant effects in human colon explants but not in epithelial cell lines, indicating possible cell- and context-dependent activity [47].

Notably, combination strategies appear more effective than pure CBD alone. In both TNBS- and DSS-induced colitis models, combined CBD and THC treatment reduced inflammatory symptoms more effectively than CBD monotherapy, with THC-mediated protection involving CB2 receptor activation [48]. Subtherapeutic combinations of THC and CBD also restored intestinal barrier integrity, reduced ammonia levels, and normalized glucagon-like peptide-1 (GLP-1) homeostasis, indicating complementary anti-inflammatory and metabolic effects [49]. Other studies have similarly shown that CBD combined with non-psychoactive cannabinoids or minor cannabis constituents can alleviate colitis-associated visceral hypersensitivity through modulation of voltage-gated sodium and calcium channels, including Cav2.2 [50,51,52,53]. In addition, combining CBD with fish oil has been reported to enhance intestinal anti-inflammatory effects at lower doses [54]. These observations support the view that full-spectrum or combination formulations may outperform isolated CBD in IBD management.

Although numerous preclinical studies have demonstrated the therapeutic potential of CBD in various IBD models, its efficacy in intestinal inflammation and precise mechanisms of action remain incompletely elucidated. CB1 receptors are widely expressed in enteric neurons and epithelial cells, whereas CB2 receptors are mainly found in immune and epithelial cells, underscoring their importance in intestinal immune regulation [55]. In Caco-2 models, CBD increased expression of the tight junction protein ZO-1 via CB1 activation and accelerated recovery of epithelial barrier integrity following EDTA or cytokine-induced disruption [56,57]. CBD also reduced S100B and iNOS expression in colon biopsies from UC patients through a PPAR-γ-dependent pathway, suggesting involvement of glial-immune signaling along the gut–brain axis [58]. Related phytocannabinoids may also be relevant; for example, cannabidivarin (CBDV) has shown anti-inflammatory effects via TRPA1 activation in experimental colitis and was associated with improved gut microbiota dysbiosis in pediatric UC, while maintaining a favorable safety profile [59].

The route of administration critically influences CBD efficacy in IBD. In TNBS-induced colitis, oral CBD failed to improve inflammation, whereas intraperitoneal and rectal delivery significantly reduced macroscopic damage, histological injury, and MPO activity [60]. These findings suggest that poor performance after oral administration may result from limited aqueous solubility, low bioavailability, and extensive first-pass metabolism. Indeed, inconsistent therapeutic outcomes of pure CBD, particularly by the oral route, remain a major challenge for translational development. To address the limitations of free CBD, advanced drug delivery systems have been explored. Lipid co-administration has been shown to increase CBD transport into intestinal lymph and enhance immunomodulatory effects [61]. Other approaches, such as biomimetic vesicles, nanoparticles, and hydrogels, aim to improve CBD solubility, stability, targeting, and local retention in inflamed intestinal tissue [62]. For instance, a CBD-loaded gelatin methacryloyl (GelMA) hydrogel showed good biocompatibility and promoted macrophage polarization toward an anti-inflammatory phenotype while reducing pro-inflammatory cytokine secretion, highlighting the potential of formulation-based optimization for IBD therapy [63].

Despite encouraging preclinical evidence, clinical data remain limited and largely inconclusive. Some studies suggest that CBD may improve intestinal barrier function, as indicated by reduced urinary lactulose/mannitol ratios in aspirin-treated subjects [64]. However, randomized controlled trials in UC and CD have not demonstrated clear improvements in objective inflammatory markers, endoscopic scores, or disease activity, although some patients reported symptomatic relief and improved quality of life [65,66]. Trials using low-dose CBD found it safe but clinically ineffective, while CBD-rich botanical extracts or CBD/THC-containing cannabis oils produced modest subjective benefits without consistent evidence of mucosal healing or biomarker reduction [67,68].

Overall, CBD represents a promising but still incompletely validated therapeutic candidate for IBD. Its anti-inflammatory, barrier-protective, immunomodulatory, and gut–brain axis-related effects are supported by substantial preclinical evidence, yet clinical translation is hindered by inconsistent efficacy, poor oral bioavailability, and variability in formulations and dosing regimens. Future research should prioritize mechanistic clarification, optimization of targeted delivery systems, and large, well-designed randomized controlled trials with standardized preparations, dose selection, and long-term follow-up to determine whether CBD can become a reliable therapeutic option for IBD.

#### 3.1.3. Gastrointestinal Cancer

Gastrointestinal (GI) cancers, including esophageal, gastric, and colorectal cancers, remain a major global health burden due to their high incidence and mortality [69]. Among phytocannabinoids, CBD has attracted growing attention as a potential therapeutic agent for GI malignancies because of its favorable safety profile, lack of psychoactivity, and broad antitumor activities. Accumulating evidence indicates that CBD exerts anticancer effects mainly by inhibiting tumor cell proliferation and inducing apoptosis and autophagy through multiple signaling pathways [70]. In vitro studies have shown that its efficacy is influenced by culture conditions, with physiologically relevant models improving translational value [71]. Although some studies reported limited effects of CBD on cell viability under standard serum conditions, others demonstrated that CBD significantly suppresses proliferation and viability of gastric and colorectal cancer cells in a dose-dependent manner [72].

Mechanistically, CBD modulates several key pathways involved in GI tumor progression. In gastric cancer, it promotes apoptosis by inducing mitochondrial dysfunction and regulating the Smac/XIAP axis [73]. In colorectal cancer (CRC), CBD has been shown to trigger reactive oxygen species (ROS) accumulation, oxidative stress, endoplasmic reticulum stress, and mitochondrial damage, ultimately leading to apoptosis through mechanisms that may be either cannabinoid receptor-dependent or -independent, depending on the cell type [74]. In oxaliplatin-resistant CRC models, CBD restored chemosensitivity by enhancing ROS production, suppressing SOD2, impairing mitochondrial function, and activating autophagy [75]. CBD can also induce G0/G1 cell-cycle arrest and apoptosis by modulating p53, ATM, p21, CDK2, cyclin E, Bax, Bcl-2, and caspase signaling [76]. In addition, it regulates the balance between autophagy and apoptosis through pathways involving Hsp70, Keap1–Nrf2, and p53 [77].

In vivo studies further support the antitumor potential of CBD. In murine colon cancer models, CBD inhibited tumor growth, angiogenesis, and metastasis, partly through downregulation of VEGF, reduction in inflammatory mediators, and enhancement of antioxidant defenses [78]. It also suppressed epithelial–mesenchymal transition (EMT) and metastatic behavior via modulation of Wnt/β-catenin signaling [79]. In carcinogen-induced colon tumorigenesis models, CBD reduced aberrant crypt foci, polyps, and tumor formation, suggesting a chemopreventive effect [80]. Related studies further showed that CBD-rich botanical formulations inhibited colon carcinogenesis and CRC cell proliferation through activation of CB1 and CB2 receptors [81].

CBD also shows promise in combination strategies, which may enhance therapeutic efficacy while reducing the toxicity of conventional treatments. It has been reported to sensitize CRC cells to TRAIL-induced apoptosis through upregulation of death receptor 5 (DR5) mediated by endoplasmic reticulum stress [82]. Transcriptomic studies identified metallothionein family genes as potential CBD-responsive targets associated with enhanced anticancer effects [83]. Synergistic activity has also been observed when CBD is combined with cisplatin, curcumin, piperine, or photodynamic therapy, with these combinations promoting apoptosis, proliferation arrest, and suppression of tumor dissemination [84,85,86]. Beyond direct tumor cell killing, emerging evidence suggests that CBD may remodel the tumor immune microenvironment by inhibiting PI3K–Akt signaling and repolarizing macrophages from an M2-like to an M1-like phenotype, thereby enhancing responsiveness to anti-PD-1 immunotherapy [87].

Despite these promising findings, the clinical translation of CBD for GI cancers remains limited, largely due to its poor oral bioavailability and unfavorable physicochemical properties [88]. Accordingly, targeted delivery systems have been developed to improve its stability and therapeutic performance. For example, a whey protein isolate-based hydrogel was shown to protect CBD from gastric degradation and facilitate effective delivery to CRC cells in vitro [89]. Overall, current evidence suggests that CBD is a multifunctional candidate for GI cancer therapy, with reported effects on tumor growth inhibition, apoptosis induction, chemosensitization, and immune modulation. However, it should be emphasized that these data are currently derived predominantly from preclinical studies, including in vitro and animal models. Controlled clinical evidence supporting the efficacy of CBD in gastrointestinal cancers remains lacking. Therefore, further studies are required to clarify its molecular mechanisms, optimize formulation and delivery strategies, and determine whether these preclinical findings can translate into clinical benefit.

### 3.2. Therapeutic Potential of CBD for Respiratory Diseases

Respiratory diseases constitute a growing global health burden, with incidence rates rising steadily due to environmental and climatic shifts, increasing air pollution, lifestyle changes, and frequent microbial infections. This trend is particularly pronounced among children and older adults, who often exhibit heightened immune vulnerability. The pathogenesis of these conditions is highly complex, driven by dynamic host–environment interactions and characterized by progressive inflammatory, structural, and functional alterations over time [90]. Although the role of CBD in respiratory diseases remains underexplored, emerging evidence suggests considerable potential in modulating immune homeostasis within the respiratory system. However, CBD exhibits context-dependent mechanisms of action, capable of either promoting or inhibiting pro-inflammatory cytokine production, highlighting the need for further mechanistic investigation.

#### 3.2.1. Inflammatory Diseases of the Respiratory System

Inflammation is a central pathological feature of a wide range of respiratory diseases, including acute lung injury (ALI), pulmonary hypertension (PH), asthma, chronic obstructive pulmonary disease, and pulmonary fibrosis, all of which contribute substantially to global morbidity and mortality [91]. Emerging evidence suggests that CBD may exert protective effects in inflammatory respiratory disorders through anti-inflammatory, antioxidant, and immunomodulatory mechanisms. In ALI models, CBD has been shown to suppress the production of pro-inflammatory cytokines such as TNF-α, IL-1β, and IL-6 in alveolar macrophages, likely via CB2-dependent inhibition of NF-κB and ERK1/2 signaling [92]. In lipopolysaccharide-induced lung injury, CBD also improved pulmonary function and attenuated inflammatory responses by reducing leukocyte infiltration, myeloperoxidase activity, and cytokine/chemokine expression, with adenosine A_2A_ receptor activation proposed as a contributing mechanism [93]. In addition, micellar CBD formulations effectively reduced neutrophil infiltration in the lung, although no enhanced efficacy was observed when combined with cannabigerol [94]. Beyond ALI, CBD has demonstrated therapeutic potential in chronic respiratory inflammation. In monocrotaline-induced PH, it reduced inflammatory mediator expression, oxidative stress, and abnormal glycolysis, while restoring mitochondrial energy metabolism [95]. In ovalbumin-induced asthma, CBD lowered levels of IL-4, IL-5, IL-6, IL-13, TNF-α, eotaxin, and IgE, thereby attenuating airway inflammation and fibrosis [96]. It also alleviated silica-induced pulmonary inflammation and fibrosis through regulation of the NLRP3/TGF-β1/Smad2/3 axis [97]. However, the immunomodulatory effects of CBD in respiratory disease are not uniformly anti-inflammatory. Some studies have reported that CBD can differentially regulate inflammatory mediators depending on cell type and pathological context, even enhancing the expression of cytokines such as IL-6, IL-8, MCP-1, TNF-α, and G-CSF, as well as inflammatory cell infiltration in bronchoalveolar lavage fluid [98]. Taken together, these findings indicate that CBD is a promising but mechanistically complex candidate for respiratory inflammatory diseases, and further studies are needed to clarify its context-dependent actions and support its rational therapeutic development.

#### 3.2.2. Coronavirus Disease-19

Coronavirus disease 2019 (COVID-19), caused by SARS-CoV-2, is a highly transmissible respiratory infection that can progress from mild symptoms to severe pneumonia, acute respiratory distress syndrome (ARDS), multi-organ failure, and death [99]. Because viral entry depends largely on angiotensin-converting enzyme 2 (ACE2) and transmembrane protease serine 2 (TMPRSS2), both highly expressed in the respiratory and gastrointestinal mucosa, these molecules have become important targets for therapeutic intervention [100]. In this context, CBD has attracted attention for its potential antiviral, anti-inflammatory, and immunomodulatory properties. Preclinical studies using 3D human tissue models and epithelial cell systems have shown that high-CBD cannabis extracts can downregulate ACE2 and TMPRSS2 expression, partly through AKT-related signaling, while also suppressing inflammatory mediators such as COX-2, IL-6, and IL-8 [101]. In addition, CBD and its metabolite 7-OH-CBD were reported to inhibit SARS-CoV-2 replication in lung epithelial cells by activating IRE1α-mediated endoplasmic reticulum stress responses and modulating interferon signaling [102]. Other studies similarly suggested that CBD-rich fractions may reduce ACE2 expression and pro-inflammatory cytokine production, although the contribution of additional cannabinoids and terpenes cannot be excluded [103]. Computational analyses and combination studies further indicate that CBD may have greater antiviral potential when used together with other bioactive compounds rather than as a single agent [104].

Beyond the respiratory tract, the intestine is also a relevant site of SARS-CoV-2 infection because of high ACE2 expression in enterocytes. In intestinal epithelial models, CBD reduced spike protein-induced inflammatory and injury-related responses by activating PPAR-γ and downregulating ACE2, RhoA-GTPase, TLR4, NLRP3, and multiple pro-inflammatory cytokines, suggesting a protective effect against virus-associated enterotoxicity [105]. CBD has also been investigated in the context of ARDS, a major cause of COVID-19 mortality driven by cytokine storm and immune dysregulation. High-CBD extracts were shown to suppress TNF-α, IL-6, and IFN-γ while increasing IL-10, reduce leukocyte migration, and attenuate systemic and pulmonary inflammation in experimental models [106]. Consistently, CBD improved inflammatory indices and clinical features in Poly I:C-induced ARDS models, supporting its potential role in mitigating cytokine storm, preserving lung structure, and restoring immune homeostasis [107]. Emerging evidence further suggests that CBD may enhance apelin signaling, which is involved in tissue repair and immune regulation, thereby providing an additional mechanistic basis for its protective effects [108].

Despite these encouraging preclinical findings, current clinical evidence remains limited and inconclusive. A small pilot placebo-controlled study suggested that sublingual CBD might improve clinical outcomes in COVID-19 patients [109]. However, a larger randomized, double-blind, placebo-controlled trial found that 300 mg/day CBD for 14 days did not significantly improve symptoms in patients with mild to moderate COVID-19 [110]. A recent systematic evaluation likewise concluded that existing evidence is insufficient to either support or refute the therapeutic repurposing of CBD for COVID-19-related inflammation and symptom control [111]. Overall, CBD shows promise as a multifunctional modulator of viral entry, host inflammatory responses, and tissue injury in COVID-19, but its clinical utility remains unproven. Further large-scale, well-controlled studies are needed to validate its efficacy, clarify discrepant findings, and define its safety and therapeutic role in COVID-19 management.

#### 3.2.3. Lung Cancer

Lung cancer remains the leading cause of cancer-related death worldwide, largely because most cases are diagnosed at advanced stages [112]. It is mainly classified into non-small cell lung cancer (NSCLC) and small cell lung cancer (SCLC), and current treatments include surgery, chemotherapy, radiotherapy, targeted therapy, and immunotherapy [113]. Despite these advances, outcomes for advanced lung cancer remain poor, highlighting the need for new therapeutic approaches. In this context, CBD has emerged as a promising candidate because of its multifaceted antitumor activities in preclinical lung cancer models [114]. CBD has been shown to induce apoptosis in NSCLC cells, suppress proliferation, reduce EGFR expression, and inhibit migration, while its combination with THC may further promote restoration of epithelial characteristics [115,116]. In addition, CBD exerts cytotoxic effects on both adherent lung cancer cells and lung cancer stem cells by activating caspases 3/7, increasing reactive oxygen species, disrupting mitochondrial membrane potential, and upregulating pro-apoptotic signaling. It also decreases cancer stem cell frequency, targets CD44, and inhibits angiogenesis, thereby suppressing tumor growth and progression [117,118].

Mechanistically, CBD appears to act through multiple molecular targets and signaling pathways, including CB1, CB2, PPAR-γ, TRPV1, p38, and p42/44 MAPK, which collectively regulate mediators such as COX-2, TIMP-1, ICAM-1, and PAI-1 and contribute to its pro-apoptotic, anti-invasive, and immunomodulatory effects [119,120,121,122,123]. Importantly, CBD may also help overcome treatment resistance, a major cause of therapeutic failure in lung cancer. It has been identified as a TRPV2-targeting compound capable of suppressing growth and metastasis in cisplatin-resistant NSCLC [124]. Moreover, combination strategies further support its therapeutic potential: CBD synergized with low-dose dasatinib by modulating the SRC/PI3K/AKT axis and enhanced the antitumor activity of cytokine-induced killer cells against NSCLC, suggesting possible utility in both targeted and immune-based combination regimens [125,126]. Overall, available data suggest that CBD exerts direct and synergistic antitumor effects in lung cancer through multi-target mechanisms involving apoptosis induction, inhibition of stemness and metastasis, and sensitization of resistant tumors. However, it should be noted that the current evidence base is still dominated by preclinical studies. Rigorous mechanistic studies and well-designed clinical trials are required to establish the therapeutic value of CBD in lung cancer.

## 4. Strategies for Enhancing the Bioavailability and Effective Delivery of CBD

**Figure 4:**
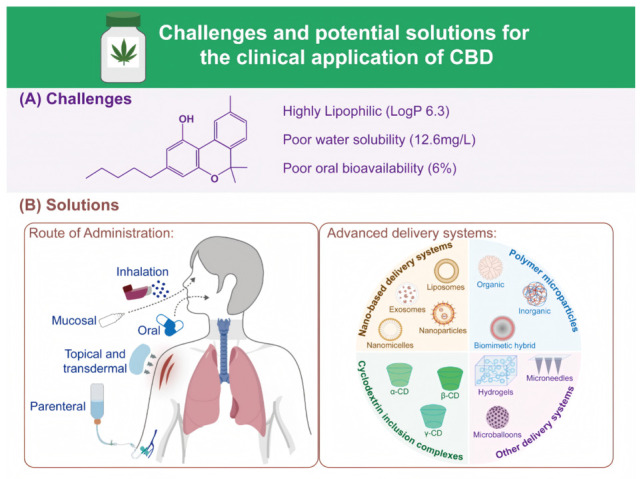
Challenges and potential solutions for the clinical application of CBD.

Although CBD has demonstrated considerable therapeutic potential in preclinical models of mucosal diseases, its translation into clinical practice remains slow, largely due to its poor bioavailability. This limitation stems not only from patient-specific physiological factors but also from the inherent physicochemical properties of CBD. Specifically, CBD is a highly lipophilic (log P = 6.3), poorly water-soluble molecule (aqueous solubility ≈ 12.6 mg/L), characteristics that qualify it as a Class II compound under the Biopharmaceutical Classification System (BCS) [127]. Moreover, CBD is susceptible to degradation under various environmental conditions, including temperature, light, and auto-oxidation [128]. The bioavailability of CBD in humans is generally low and highly variable. Reported values differ substantially across studies depending on formulation, dose, fed or fasted state, route of administration, and study design. In particular, the limited and inconsistent systemic exposure observed after oral administration is largely attributed to poor aqueous solubility, incomplete gastrointestinal absorption, and extensive first-pass metabolism in the liver [129]. These inherent properties also hinder the development of stable, precisely targeted formulations capable of achieving effective drug delivery to disease-specific sites. Consequently, optimizing the route of administration and designing advanced delivery systems appear essential to overcoming the bioavailability and targeting challenges associated with CBD (Figure 4).

### 4.1. Route of Administration

CBD can be administered via multiple routes, including oral, mucosal, inhalation, injection, and transdermal delivery. Bioavailability differs markedly across administration routes, but these estimates should be interpreted with caution because they are highly study-dependent. In general, oral CBD shows low and variable bioavailability, whereas oromucosal and inhaled administration may provide higher systemic exposure in some studies [130,131]. However, the reported values vary considerably according to formulation characteristics, dosing conditions, delivery devices, and pharmacokinetic study design. Therefore, the selection of an optimal administration route is critical for achieving desired therapeutic outcomes. This requires a comprehensive consideration of CBD’s physicochemical properties, the pathophysiology of the target disease, and the comparative pharmacokinetic profiles associated with each delivery pathway.

#### 4.1.1. Oral Administration

Oral administration represents the most common, convenient, and widely accepted route of drug delivery [132]. Although oral CBD is generally well-tolerated, considerable inter- and intra-individual variability in its pharmacokinetics has been observed. Additionally, CBD undergoes rapid and extensive metabolism to 7-COOH-CBD, contributing to its low absolute bioavailability [133]. The significant first-pass effect further limits the clinical and commercial utility of orally administered CBD. Co-administration with food—particularly high-fat or high-calorie meals—can enhance the bioavailability of CBD by approximately fourfold [134]. This effect is attributed to improved solubilization of the highly lipophilic compound, delayed gastric emptying, stimulated bile secretion, and modified intestinal metabolism. Consequently, dietary fat intake can reduce fluctuations in systemic exposure and substantially increase bioavailability, supporting the recommendation to administer CBD with meals for optimal absorption [135]. Based on this principle, an oil-based oral CBD solution has successfully completed clinical trials and advanced to commercialization. Epidiolex^®^—developed by GW Pharmaceuticals—is the first FDA-approved purified CBD drug, indicated for epilepsy associated with Dravet syndrome and Lennox–Gastaut syndrome in patients aged two years and older [136]. It is a strawberry-flavored oral solution with sesame oil as the primary excipient; thus, it is contraindicated in patients with sesame allergies. The FDA also recommends assessing liver function tests before initiation and during treatment. Beyond conventional oil formulations, CBD lipid-based delivery systems have attracted widespread interest for their potential to reduce first-pass metabolism, promote lymphatic transport, and improve oral bioavailability [137]. While these approaches currently represent the most viable strategies for enhancing CBD absorption, they still face several challenges. For example, lipid formulations may undergo gastrointestinal digestion, leading to drug precipitation and limited intestinal absorption. CBD oils also exhibit variable absorption, resulting in unpredictable pharmacokinetics, and certain carrier oils carry allergy risks [138]. Therefore, developing novel and optimized oral CBD formulations remains highly necessary. In a single-center, open-label, randomized trial, PTL101—an oral formulation comprising CBD-loaded gelatin microparticles in enteric capsules—demonstrated favorable safety and tolerability. Compared with a commercial CBD oromucosal spray, PTL101 (10 mg) showed 1.7- and 1.3-fold higher Cₘₐₓ and AUC, respectively, with a relative bioavailability of 134% [139]. In another randomized, double-blind, placebo-controlled study, a novel CBD capsule (TurboCBD™) exhibited excellent tolerability in healthy male volunteers and achieved 111% higher bioavailability compared to a conventional 90 mg oral CBD formulation [140].

#### 4.1.2. Mucosal Administration

Mucosal tissues, including the oral, nasal, rectal, and vaginal mucosae, represent highly attractive routes for CBD delivery because of their large surface area, rich vascularization, high permeability, and ability to partially or fully bypass hepatic first-pass metabolism [141]. These advantages support noninvasive administration, reduced dosing frequency, and improved patient compliance. The clinical success of Sativex^®^, an oromucosal spray containing CBD and THC, has further stimulated interest in mucosal CBD formulations [142]. Among oral mucosal approaches, buccal delivery is particularly suitable for sustained release because of the relative immobility of the tissue, whereas sublingual delivery enables faster systemic absorption owing to its thin and highly vascularized structure [143]. Advanced formulations such as nanoemulsions, cyclodextrin complexes, 3D-printed oral films, and bigels have demonstrated improved CBD release, absorption, palatability, and dose personalization, although salivary clearance and relatively slow mucosal permeation remain important limitations for oromucosal sprays [144,145,146].

Intranasal delivery has emerged as a particularly promising strategy because it combines rapid systemic absorption with the potential for direct nose-to-brain transport, thereby enhancing bioavailability and central nervous system targeting [147]. Nanoemulsions and thermosensitive hydrogels have significantly increased CBD exposure and shortened absorption time compared with oral delivery, supporting their potential for neurological and psychiatric indications [148]. Rectal administration, although limited by smaller mucosal surface area, remains valuable for both local and systemic therapy, especially in patients unable to tolerate oral medications. Transferosome-based systems and 3D-printed hollow suppositories have improved colorectal permeation, formulation flexibility, and patient acceptability [149,150]. Vaginal and related urogenital applications of CBD remain less explored but show emerging promise for local anti-inflammatory and antimicrobial therapy. CBD-containing microcapsules, nanoparticles, and 3D-printed hydrogel scaffolds have demonstrated antibacterial, antioxidant, and sustained-release properties, suggesting potential utility in vaginal inflammation and urinary tract infections [151,152,153]. Overall, mucosal delivery offers a versatile and clinically relevant platform for overcoming the biopharmaceutical limitations of CBD, although formulation optimization and further translational validation are still required.

#### 4.1.3. Inhalation Administration

Inhalation represents a highly attractive route for CBD delivery because it is noninvasive and enables rapid systemic absorption, high bioavailability, and improved dose titration [154]. Compared with oral administration, inhaled CBD consistently exhibits markedly faster absorption and greater systemic exposure. In preclinical studies, inhaled CBD achieved a substantially shorter T_max_ and significantly higher C_max_ and AUC than oral CBD, although high inhaled doses were associated with dose-dependent respiratory tissue changes, whereas exposure levels relevant to typical human use appeared well tolerated [155,156]. Clinical studies further support these pharmacokinetic advantages. Inhaled CBD produced measurable subjective effects without significant cognitive or somatic impairment, while oral CBD showed little difference from placebo in comparable settings [157]. Moreover, a Phase I trial demonstrated that a dry powder inhaler (DPI) formulation of CBD achieved much faster absorption and markedly higher peak plasma concentrations than Epidiolex^®^ oral solution, confirming the superior bioavailability of pulmonary delivery [158]. To optimize inhaled CBD formulations, several powder-based systems have been developed. These include high-drug-load powders prepared by wet ball milling, spray-dried formulations using hydroxypropyl-β-cyclodextrin as a solubilizer, and nano-hydrate suspensions generated by nanoprecipitation. Such systems achieved favorable aerosolization characteristics, including high fine particle fractions and appropriate aerodynamic diameters, while also demonstrating acceptable biocompatibility in lung cell models [159,160,161]. At the same time, the increasing use of CBD-containing electronic cigarettes has raised important safety concerns. Although e-cigarette vapor effectively delivers CBD and produces dose-dependent plasma exposure, preclinical studies indicate that it may also induce hypothermia, reduce locomotor activity, and exert greater cytotoxic and pro-inflammatory effects on human cells than nicotine-containing products, with more pronounced lung injury-related pathological changes [162,163]. Overall, inhalation offers a promising strategy to overcome the poor oral bioavailability of CBD and achieve rapid therapeutic exposure, but formulation design and long-term pulmonary safety require careful evaluation before broader clinical translation.

#### 4.1.4. Parenteral Administration

Parenteral administration, including intravenous, intramuscular, and subcutaneous injection, provides an alternative route for CBD delivery that avoids gastrointestinal degradation and first-pass metabolism while enabling rapid onset and high systemic exposure [164]. Pharmacokinetic studies across species consistently demonstrate the favorable disposition of parenteral CBD, with markedly higher bioavailability and plasma exposure than oral administration [165]. In comparative studies, intravenous CBD produced substantially higher C_max_ and AUC values and a much shorter T_max_ than oral dosing, while maintaining comparable pharmacodynamic efficacy, highlighting its superiority in achieving rapid and efficient systemic delivery [166]. Intramuscular administration has also shown promise, particularly for chronic pain management, and CBD nanocrystal formulations further improved absorption compared with oral formulations and oil-based preparations [167]. In addition, subcutaneous liposomal CBD enabled sustained drug release over several days, suggesting potential utility for prolonged analgesic therapy, such as in osteoarthritis [168]. Beyond pharmacokinetic advantages, injectable CBD has demonstrated therapeutic potential in multiple preclinical models, including depression, neurological disorders, compulsive behavior, and ischemia/reperfusion-induced liver injury, with its pharmacodynamic effects generally correlating with systemic exposure profiles [169,170,171,172,173,174]. These findings indicate that parenteral delivery may be particularly valuable in clinical settings requiring rapid, reliable, or sustained CBD exposure. However, despite these advantages, the broader application of parenteral CBD remains constrained by concerns related to injection-associated safety, cost, invasiveness, and limited practicality for long-term use. In summary, parenteral administration represents an effective strategy to maximize CBD bioavailability and therapeutic exposure, but further investigation is needed to define its optimal formulations and clarify its role in specific clinical indications.

#### 4.1.5. Topical and Transdermal Administration

The skin is an attractive route for CBD delivery because it is noninvasive, bypasses gastrointestinal and hepatic first-pass metabolism, enables sustained drug input, and generally improves patient compliance [175]. However, effective dermal and transdermal delivery of CBD remains challenging because of the barrier function of the stratum corneum and the high lipophilicity of CBD, which favors its retention in the outer skin layers while limiting penetration into deeper tissues and systemic circulation [176]. To address these limitations, multiple formulation strategies have been developed, including penetration enhancer-containing gels, microemulsions, organosilica-based films, nanoemulsions, transdermal patches, and microneedle systems [177,178,179,180,181]. These approaches have generally improved CBD stability, skin permeation, retention, and systemic exposure, with microneedle-based delivery showing particularly promising pharmacokinetic performance for systemic administration. Available preclinical and early clinical evidence further suggests that topical and transdermal CBD may have therapeutic value in chronic inflammatory and neurological conditions, such as arthritis and epilepsy [182,183]. In pediatric patients with developmental and epileptic encephalopathy, transdermal CBD gel showed favorable tolerability and was associated with reduced seizure frequency and burden [184]. Likewise, exploratory human data confirmed that CBD can penetrate the skin and reach systemic circulation following transdermal administration [185]. Nevertheless, the intrinsic physicochemical properties of CBD, together with lag time in systemic absorption and fixed dosing constraints, continue to limit the efficiency and flexibility of transdermal delivery. Taken together, dermal and transdermal systems represent promising strategies for CBD administration, particularly for chronic and localized indications, although further formulation optimization and clinical validation are still required.

### 4.2. Advanced Delivery Systems

The primary objective of a drug delivery system is to achieve precise spatiotemporal control over drug release, ensuring optimal concentrations at the target site [186]. Conventional formulations—such as tablets, capsules, and ointments—often suffer from limitations including systemic adverse effects and poor bioavailability. To overcome these challenges, advanced drug delivery systems have emerged, integrating innovative technologies and novel formulations to enhance targeting and release profiles. These systems substantially improve therapeutic efficacy, safety, and patient compliance [187]. This is particularly relevant for Biopharmaceutics Classification System (BCS) Class II drugs like CBD, where such platforms not only increase solubility but also enable controlled release, thereby significantly improving bioavailability (Table 1).

#### 4.2.1. Nano-Based Delivery Systems

Nano-based delivery systems have emerged as promising platforms for CBD delivery because they can improve targeting, prolong circulation, enhance biocompatibility, reduce systemic toxicity, and overcome the poor solubility and low bioavailability of free CBD [204]. Among these systems, liposomes and niosomes are widely used vesicular carriers with favorable encapsulation capacity and delivery performance. Liposomal CBD has been shown to improve dissolution and antitumor activity, whereas niosomal formulations exhibited superior regulation of inflammatory and apoptotic biomarkers compared with free CBD [205,206]. Exosomes, as naturally derived nanovesicles, offer additional advantages including prolonged half-life, high biocompatibility, and intrinsic targeting potential. Functionalized exosome-based CBD systems have demonstrated therapeutic efficacy in neurological disease models, particularly through attenuation of neuroinflammation and modulation of disease-related signaling pathways [207,208].

Nanoparticles and polymer-based nanocarriers have also shown substantial potential for enhancing CBD delivery. Protein-based and polysaccharide-based nanoparticles improved CBD stability, antioxidant activity, and release behavior, while targeted nanoparticle systems enabled tissue-specific delivery and enhanced therapeutic efficacy in inflammatory and oncological models [209,210]. In particular, oral colon-targeting nanoparticles restored mucosal barrier integrity, modulated the TLR4–NF-κB pathway, and improved gut microbiota composition in ulcerative colitis models, whereas adhesive polymeric nanoparticles increased encapsulation efficiency and antitumor activity in bladder cancer [211,212]. Polymeric nanomicelles and nanogels further expand the delivery toolbox for CBD by providing efficient solubilization of hydrophobic molecules and controlled release. These systems have shown improved anti-inflammatory efficacy, enhanced biosafety, and favorable permeability in applications such as oral mucositis and corneal delivery [213,214,215].

Self-nanoemulsifying drug delivery systems (SNEDDS) are another important strategy for oral CBD administration. By spontaneously forming nanoemulsions in the gastrointestinal tract, SNEDDS can enhance intestinal absorption, promote lymphatic transport, and partially bypass first-pass metabolism [216]. Recent pharmacokinetic studies demonstrated that CBD-SNEDDS achieved faster absorption and greater systemic exposure than conventional oil-based formulations and even commercial oral preparations [217]. Novel excipient systems, such as therapeutic deep eutectic solvent-based SEDDS, have further broadened the formulation design space while maintaining stability and permeability [218].

Taken together, nano-delivery systems markedly improve the biopharmaceutical performance of CBD and have shown encouraging therapeutic effects in diverse preclinical models. However, whether improved pharmacokinetics consistently translates into superior clinical efficacy remains unclear. In addition, the long-term safety of nanocarriers requires careful evaluation, as these systems may alter CBD biodistribution, metabolism, and excretion or introduce carrier-related toxicities. Therefore, although CBD-loaded nanoformulations hold considerable translational promise, further rigorous clinical studies are needed to clarify their efficacy, safety, and patient acceptability.

#### 4.2.2. Polymer Microparticles

Polymer microparticles (MPs) have emerged as promising carriers for CBD delivery because they enable controlled release, protect labile compounds, and can be tailored in terms of morphology, size, and polymer compositions [219]. In particular, biodegradable polymer-based MPs, especially core–shell systems, are well suited for encapsulating hydrophobic molecules such as CBD. Recent studies have shown that poly(ε-caprolactone) (PCL) MPs can provide sustained CBD release for up to 10 days after a single administration, supporting their potential as long-acting delivery systems [220]. In tumor models, CBD-loaded PCL MPs enhanced apoptosis, inhibited proliferation and angiogenesis, and achieved antitumor effects comparable to repeated administration of free CBD solution, highlighting their value for improving therapeutic persistence while reducing dosing frequency [221,222]. Polymer MPs also offer advantages in combination therapy. CBD-loaded MPs have been shown to enhance the antiproliferative activity of conventional chemotherapeutic agents such as paclitaxel and doxorubicin, indicating synergistic effects that may allow dose reduction and reduced toxicity [223,224]. Beyond oncology, injectable biomimetic hydrogel microparticles loaded with CBD have demonstrated uniform morphology, favorable physicochemical properties, and therapeutic potential in tissue repair, as evidenced by improved neural regeneration and functional recovery in a sciatic nerve injury model [225]. These findings underscore the versatility of polymer MPs for both local and sustained CBD delivery across diverse indications. However, the performance of polymer MPs is highly dependent on formulation parameters, including particle morphology, size, molecular weight, terminal functional groups, and polymer–medium interactions, all of which influence degradation behavior and drug release kinetics [226]. In addition, many conventional fabrication processes rely on toxic organic solvents, raising concerns regarding safety, environmental impact, and manufacturing sustainability [227]. Taken together, polymer microparticle systems represent a highly promising platform for long-acting and targeted CBD delivery, but further optimization of material selection, processing methods, and translational evaluation is required to fully realize their clinical potential.

#### 4.2.3. Cyclodextrin Inclusion Complexes

Cyclodextrins (CDs) are cyclic oligosaccharides with a hydrophilic external surface and a hydrophobic inner cavity, enabling them to form inclusion complexes with lipophilic compounds such as CBD and thereby improve its aqueous solubility, physicochemical stability, and bioavailability [228]. Native CDs, including α-, β-, and γ-CD, as well as chemically modified derivatives, have been widely investigated as carriers for CBD. Existing studies show that CBD can be stably incorporated into CD cavities, resulting in markedly enhanced water solubility and, in some cases, improved antioxidant, cytotoxic, and antitumor activities [229,230]. In particular, modified CDs such as DM-β-CD and bridged CD dimers have demonstrated substantially greater solubilizing capacity than native CDs, while maintaining favorable biocompatibility [231,232]. Functionalized CD systems have also been developed to provide additional advantages, including tumor-targeting capability and pH-responsive release behavior [233]. Beyond simple inclusion complexes, CD-based strategies have increasingly been integrated with advanced delivery technologies. Nanoformulations incorporating CBD–CD complexes have shown markedly improved solubility, stability, permeation, and therapeutic performance, as exemplified by intranasal nanomicelles for inflammatory conditions and cyclodextrin–lipid nanofiber systems for brain-targeted delivery [234,235]. In parallel, the combination of CD inclusion technology with 3D printing has enabled the fabrication of personalized oral formulations with controlled and site-specific release, including colon-targeted CBD delivery systems [236]. Taken together, cyclodextrin-based formulations represent a versatile and effective platform for overcoming key biopharmaceutical limitations of CBD [237]. However, despite encouraging preclinical results, further studies are still needed to optimize formulation design, validate therapeutic advantages in vivo and clinically, and expand their application in precision medicine and complex disease management.

#### 4.2.4. Other Delivery Systems

Beyond the delivery systems discussed above, hydrogel-based platforms have emerged as particularly promising carriers for CBD owing to their high water content, tunable physicochemical properties, excellent biocompatibility, and capacity to protect unstable drugs while enabling sustained release [238]. Recent studies have demonstrated the versatility of CBD-loaded hydrogels across multiple biomedical applications. For example, whey protein isolate hydrogels promoted pre-osteoblast proliferation and osteogenic activity, suggesting potential in bone tissue regeneration [239]. Hydrogel-based microneedle patches and injectable in situ gels have also shown significant therapeutic effects in psoriasis and spinal cord injury models, respectively, by enabling localized and sustained CBD delivery and modulating inflammation-, apoptosis-, and regeneration-related pathways [240,241]. In ophthalmic applications, CBD-loaded hydrogel contact lenses have further exhibited multifunctional effects, including pH regulation, anti-infection, anti-angiogenesis, and corneal repair [242]. Hybrid systems combining hydrogels with nanocarriers appear particularly advantageous for dermal delivery, as they improve the loading of hydrophobic CBD while overcoming the low viscosity and limited applicability of standalone nanocarriers [243,244]. In addition, alternative carriers such as carbon xerogel microspheres and Pickering emulsions have shown potential for sustained or localized CBD delivery [245,246]. Nevertheless, the hydrophilic nature of hydrogels remains a major challenge for efficient encapsulation of lipophilic molecules such as CBD, necessitating formulation strategies that introduce hydrophobic domains or functional components into the network. Taken together, hydrogel-based and related hybrid delivery systems provide a versatile platform for topical, transdermal, injectable, and implantable CBD administration. Further optimization of carrier design, formulation strategy, and disease-specific validation will be essential to fully realize their translational and clinical potential.

#### 4.2.5. Comparative Perspective and Translational Prioritization

Not all CBD-based delivery systems are equally close to practical therapeutic application. In general, platforms that primarily address the major biopharmaceutical limitations of CBD while relying on relatively simple composition, scalable manufacturing, and familiar regulatory pathways appear more translationally feasible in the near term. These include lipid-based oral formulations, cyclodextrin-enabled systems, mucoadhesive mucosal dosage forms, and transdermal or topical formulations, which may improve solubility, local retention, or systemic exposure without introducing excessive formulation complexity. Such systems are particularly attractive when the therapeutic goal is to enhance bioavailability or achieve local delivery at accessible mucosal sites. By contrast, more sophisticated platforms, such as highly engineered nanoparticles, exosome-based systems, multifunctional hybrid carriers, and some injectable or implantable formulations, may offer advantages in targeting, controlled release, or tissue-specific delivery, but their translational path is less straightforward [247]. These systems often face additional challenges related to large-scale manufacturing, batch-to-batch reproducibility, carrier-associated safety, cost, and regulatory evaluation. Polymer microparticles may be especially promising for sustained local delivery, but their performance is strongly formulation-dependent and may be limited by processing complexity. Hydrogel-based systems are highly versatile and well suited for local applications, although loading hydrophobic CBD efficiently remains challenging. Overall, the most promising platform should be selected according to the intended route, disease site, required exposure profile, and translational readiness, rather than assuming that greater formulation sophistication necessarily leads to greater clinical utility.

## 5. Challenges and Future Directions

Despite the broad therapeutic promise of CBD in mucosal diseases, its clinical translation remains challenging. The current evidence base is still dominated by preclinical studies, whereas convincing and reproducible clinical benefit has not yet been consistently demonstrated across indications. In addition to its intrinsic biopharmaceutical limitations, such as poor aqueous solubility, low and variable bioavailability, and extensive first-pass metabolism, several broader issues continue to hinder translation, including the gap between preclinical and clinical outcomes, unresolved safety concerns, the limited clinical translatability of advanced delivery systems, and the fragmented regulatory landscape surrounding CBD-containing products. Accordingly, future progress in this field will require not only more sophisticated formulations, but also a more cautious and evidence-based framework for safety evaluation, regulatory positioning, and clinical development.

### 5.1. Why Promising Preclinical Findings Have Not Consistently Translated into Clinical Benefit

Although CBD has shown encouraging therapeutic effects in multiple preclinical models of mucosal diseases, these findings have not consistently translated into clear clinical benefit. Several factors may contribute to this translational gap. First, many preclinical studies use conditions that maximize CBD exposure, whereas clinical studies often rely on oral or other patient-friendly formulations limited by poor aqueous solubility, low and variable bioavailability, and extensive first-pass metabolism. As a result, effective concentrations observed in experimental systems may not be reliably achieved in humans. Second, preclinical models do not fully reflect the complexity and heterogeneity of human mucosal diseases, which are influenced by chronic inflammation, comorbidities, concomitant medications, and interindividual variability. Third, substantial heterogeneity exists across CBD formulations, including purified CBD, botanical extracts, and combination products, which differ in pharmacokinetics and pharmacological effects. In addition, preclinical studies often assess mechanistic or biomarker-based outcomes, whereas clinical trials typically evaluate broader symptomatic or composite endpoints, making translation more difficult. Finally, current clinical studies remain limited by small sample sizes, short treatment duration, variable dosing regimens, and insufficient standardization. Together, these factors may explain why promising preclinical results have not yet produced consistent clinical efficacy. Future research should therefore emphasize clinically relevant models, optimized and standardized formulations, exposure-matched study designs, and well-powered randomized controlled trials.

In oncology, preliminary human observations remain extremely limited. Isolated case reports have described apparent tumor regression following self-administration of CBD-containing preparations in lung cancer patients who did not receive standard therapy [248,249]. However, such reports are anecdotal and hypothesis-generating only, and should not be interpreted as evidence of clinical efficacy. Their clinical relevance requires validation in well-designed controlled studies.

### 5.2. Safety Pharmacology of CBD

Safety remains an important consideration for the clinical translation of CBD. Although CBD is often regarded as generally well tolerated, clinically relevant adverse effects and drug–drug interactions have been documented, particularly with purified pharmaceutical formulations. Hepatotoxicity is one of the best recognized concerns, with liver enzyme elevations showing dose dependence and being more frequent in patients receiving concomitant medications such as valproate and clobazam. In addition, CBD can inhibit cytochrome P450 enzymes and alter the exposure of co-administered drugs, highlighting the risk of clinically significant pharmacokinetic interactions [250,251]. These findings indicate that the safety profile of CBD is not only dose-dependent but also strongly influenced by formulation and co-medication status. Therefore, future clinical studies in mucosal diseases should incorporate careful safety pharmacology assessment, including liver function monitoring and evaluation of potential drug–drug interactions.

### 5.3. Limitations in the Clinical Translatability of Advanced CBD Delivery Systems

Although advanced CBD delivery systems have shown considerable promise in improving solubility, stability, targeting, and release behavior, their immediate clinical applicability should not be overstated. Most available evidence is still derived from in vitro studies or animal models, and improved delivery performance does not necessarily translate into superior clinical efficacy. In addition, some carriers may alter the biodistribution, metabolism, or long-term tissue exposure of CBD, creating safety concerns that are not fully captured by short-term preclinical studies. Increasingly sophisticated formulations may also face practical barriers, including difficulties in large-scale manufacturing, batch-to-batch reproducibility, cost control, and regulatory approval. Accordingly, the development of advanced CBD formulations should be guided not only by improvements in pharmaceutical performance, but also by long-term safety, translational feasibility, and manufacturability.

### 5.4. Regulatory Status and Oversight Considerations

The regulatory landscape of CBD remains fragmented and highly jurisdiction-dependent. A distinction should be made between pharmaceutical-grade CBD products approved as medicines and non-licensed consumer CBD products, which often differ substantially in quality, consistency, and regulatory oversight. At present, approved drug products containing purified CBD are limited to a small number of indications in selected regions, whereas most other CBD-containing products remain outside harmonized pharmaceutical regulation. This situation creates major translational challenges, including inconsistent legal definitions, variable product standards, and uncertainty regarding purity, batch-to-batch consistency, and labeling accuracy. For the development of CBD-based therapies for mucosal diseases, regulatory approval will require not only demonstration of efficacy and safety, but also robust quality control, manufacturing standardization, and indication-specific clinical evidence.

### 5.5. Emerging Technologies and Future Formulation Strategies

Emerging technologies may offer additional opportunities for future CBD formulation development, but their current relevance should be interpreted cautiously. Direct applications of AI-guided formulation design to CBD remain limited, and its role in this field is still largely prospective [252]. By contrast, 3D printing has already been explored in several CBD-related dosage forms, including oral films, hollow suppositories, hydrogel-based scaffolds, and colon-targeted formulations. Nevertheless, the use of 3D printing for truly personalized CBD therapy remains at an early stage and requires further validation in terms of formulation robustness, manufacturability, and clinical relevance [253].

Most studies discussed in this review involved highly purified CBD. However, some investigators have proposed that combinations of CBD with other cannabis constituents may produce additional therapeutic effects through the so-called “entourage effect” [254]. Mechanistically, this concept has been attributed to possible pharmacodynamic complementarity among cannabinoids and terpenes, and potentially to pharmacokinetic influences on absorption, distribution, or metabolism. Nevertheless, the mechanistic basis remains unresolved and the available evidence is inconsistent. While phytocannabinoid–terpenoid synergy has been proposed in the literature, subsequent receptor-level studies reported that common cannabis terpenes did not measurably alter THC activity at CB1 or CB2 receptors [255]. In addition, an in vitro comparison of pure CBD and CBD oils found that pure CBD was as potent as, or more potent than, the most active oil preparation tested, arguing against a universal entourage effect [256]. Therefore, whether purified CBD or full-spectrum extracts are preferable should be considered indication-specific and evidence-driven, rather than assuming the inherent superiority of either approach.

### 5.6. Future Directions

Taken together, the future development of CBD for mucosal diseases should move beyond proof-of-concept efficacy and place greater emphasis on translational rigor. Priorities should include the use of clinically relevant disease models, better alignment between preclinical exposure and human dosing, standardized and well-characterized formulations, biomarker-guided evaluation, and appropriately powered randomized controlled trials. At the same time, long-term safety pharmacology, carrier-associated toxicity, and manufacturing feasibility should be incorporated earlier in development. Only through the integration of pharmacology, formulation science, clinical methodology, and regulatory strategy can CBD-based interventions be advanced from promising experimental tools to safe, effective, and clinically applicable therapies for mucosal diseases.

## 6. Conclusions

In summary, CBD demonstrates clear therapeutic potential in the treatment of mucosal diseases. Advances in understanding its molecular targets and pharmacological mechanisms are gradually elucidating its pathways of action, providing a theoretical basis for precision therapy. However, multiple challenges remain for its clinical translation and commercialization. Beyond its inherent physicochemical limitations, issues such as insufficient preclinical data, a lack of high-quality randomized controlled trials, evolving regulatory frameworks, and persistent public misconceptions must be addressed. To fully realize the therapeutic promise of CBD, rational selection of administration routes and the development of novel delivery systems are crucial. Moreover, the integration of emerging technologies—such as artificial intelligence and 3D printing—holds promise for accelerating dosage form design and personalized treatment strategies, thereby shortening the path from bench to bedside. We encourage more researchers to engage in this field and collectively advance both fundamental and translational studies on CBD for mucosal disorders. Only through rigorous scientific validation, leading to the development of safe, effective, and quality-controlled CBD formulations, can broad medical acceptance and public trust be achieved—ultimately benefiting patients worldwide.

## Figures and Tables

**Table 1:** Summarized information on advanced delivery systems/formulations of CBD.

Delivery System/Formulation	API	Route of Administration	Performance	Application	Ref.
Nanoparticles	CBD	Mucosal	Anti-Inflammatory	Neurological	[188]
	CBD and ApoE	Mucosal	Anti-Inflammatory and clear Aβ plaques	Alzheimer’s disease	[189]
	CBD	Transdermal	Anti-Inflammatory	Dermal diseases	[190]
Exosomes	CBD	Oral	Antiproliferative and anti-Inflammatory	Lung Cancer	[191]
	CBD	Oral	Enhance brain-targeting and efficacy	Neurological	[192]
Nanoemulsion	CBD	Oral	Enhanced bioavail ability	-	[193]
Hydrogels	CBD	Transdermal	Antibacterial, antibacterial and anti-Inflammatory	Wound dressing	[194]
	CBD	Topical	Antioxidant and anti-inflammatory	Combined radiation and wound skin injury	[195]
	CBD and TER	Transdermal	Antimicrobial, anti-inflammatory, antioxidant, and anti-aging	Dermal diseases	[196]
	CBD	Transdermal	Anti-bacterial, anti-inflammation, angiogenic and osteogenic	Repair open bone defects	[197]
	CBD	Transdermal	Antioxidant, anti-inflammatory, and analgesic	Oral mucosal lesions	[198]
HPMC-PVA film	CBD	Mucosal	Enhanced bioavailability	Neurological	[199]
Oral spray	CBD	Mucosal	Anti-Inflammatory	Oral ulcer	[200]
Nanostructured lipid carriers	CBD	Mucosal	Brain targeting	Epilepsy	[201]
	CBD	Mucosal	Antinociceptive	Neuropathic pain	[202]
	CBD	Topical and transdermal	Chemical stability and Anti-Inflammatory	Dermal diseases	[203]

CBD, cannabidiol; API, active pharmaceutical ingredient; Ref, reference; ApoE, Apolipoprotein E; TER, α-terpineol; HPMC, hydroxypropyl methylcellulose; PVA, polyvinyl alcohol.

## Data Availability

Data sharing is not applicable to this article as no new data were created or analyzed in this study.
